# Drug-Eluting Balloons versus Second-Generation Drug-Eluting Stents for Treating In-Stent Restenosis in Coronary Heart Disease after PCI: A Meta-Analysis

**DOI:** 10.1155/2018/7658145

**Published:** 2018-07-24

**Authors:** Wen-Juan Xiu, Hai-Tao Yang, Ying-Ying Zheng, Yi-Tong Ma, Xiang Xie

**Affiliations:** Heart Center, First Affiliated Hospital of Xinjiang Medical University, Urumqi, Xinjiang 830011, China

## Abstract

**Background:**

In-stent restenosis (ISR) remains a common problem following percutaneous coronary intervention (PCI). However, the best treatment strategy remains uncertain. There is some controversy over the efficacy of drug-eluting balloons (DEBs) and second-generation drug-eluting stents (DESs) for treating ISR.

**Methods:**

A meta-analysis was used to compare the efficacy of the DEB and second-generation DES in the treatment of ISR. The primary endpoint is the incidence of target lesion revascularization (TLR). The secondary endpoint is the occurrence of target vessel revascularization (TVR), myocardial infarction (MI), all-cause death (ACM), cardiac death (CD), major adverse cardiac events (MACEs), minimum luminal diameter (MLD), late luminal loss (LLL), binary restenosis (BR), and percent diameter stenosis (DS%).

**Results:**

A total of 12 studies (4 randomized controlled trials and 8 observational studies) including 2020 patients with a follow-up of 6–25 months were included in the present study. There was a significant difference in the MLD between the two groups during follow-up (*P*=0.007, RR = 0.23, and 95% CI: 0.06–0.4 mm). There was no significant difference in LLL, BR, or DS% and the overall incidence of MACEs between the two groups. Subgroup analysis showed no significant difference in the incidence of primary and secondary endpoints when considering RCTs or observational studies only.

**Conclusions:**

The efficacy of the DEB and second-generation DES in the treatment of ISR is comparable. However, our results need further verification through multicenter randomized controlled trials.

## 1. Introduction

Over the past few decades, an exponential increase in percutaneous coronary intervention (PCI) has led to a significant improvement in the clinical outcomes of coronary artery disease (CAD) patients. PCI has also been widely adopted as part of the standard treatment for CAD. However, in-stent restenosis (ISR) has become one of the main problems affecting the prognosis of patients after PCI, especially for complex diseases such as chronic occlusive disease or calcification or in patients with diabetes mellitus and chronic renal insufficiency [1–3]. Studies have shown that the incidence of ISR (BMS-ISR) is as high as 16–44% after implantation of bare-metal stents (BMSs) [4]. The first generation of the DES (sirolimus DES and paclitaxel DES) used permanent materials for coating, which increased the risk of advanced and late-stage thrombosis [5]. The incidence of ISR (DES-ISR) is as high as 5–15% [4]. A new generation of the DES uses a different stent framework material, new antiproliferative drugs (including biolimus, everolimus, and zotarolimus), and biodegradable materials for coating compared to the first generation (including cobalt chromium alloy and platinum chromium alloy) of the DES. Due to its improved biocompatibility and thinner stent beam, the new generation of the DES will result in earlier endothelialization, reducing the incidence of neointimal hyperplasia, restenosis, and late and very late stent thrombosis [5]. China's I-LOVE-IT 2 study [6] showed that the target lesion failure rate in the new generation of the biodegradable coating DES was not inferior to that of the permanent coating DES within 1 year of follow-up. Furthermore, the efficacy and safety of the DES with a biodegradable coating after 6 months of dual antiplatelet therapy (DAPT) were not inferior to those after 12 months of DAPT [7]. The DEB releases antirestenosis drugs in local lesions through the balloon surface during dilation for treatment. DEBs are recommended for the treatment of restenosis with BMSs or DESs [8, 9]. Currently, DEBs may be considered to be the preferred treatment regimen for patients with restenosis associated with BMSs and DESs, particularly in patients with multiple stents, large branching lesions, and DAPT intolerance [5]. Additionally, the efficacy of the DEB has been demonstrated in both randomized controlled trials and real-world scenarios [10]. Previous studies have suggested that, for the treatment of ISR, the DEB is superior to plain old balloon angioplasty (POBA) but is not inferior to the DES [11, 12]. However, many studies have compared the effectiveness and safety between the DEB and first-generation DES [10]. The second-generation DES, such as the everolimus-eluting stent (EES), has been widely used due to the lower incidence of target vessel revascularization and stent thrombosis [13]. Many studies have reported the comparative effectiveness and safety between the DEB and second-generation DES [14–24], but the results remain controversial. To further explore the efficacy of the DEB and second-generation DES, we searched the recent literature to perform a meta-analysis.

## 2. Materials and Methods

The inclusion and exclusion criteria were in accordance with the Cochrane Handbook for Systematic Reviews manual [26].

### 2.1. Inclusion Criteria

The inclusion criteria for this study are as follows:

1. *Subjects*: the original study clearly articulated that the subjects met the diagnostic criteria of coronary artery ISR, including BMS-ISR and DES-ISR.
2. *Number of patients included in the study*: at least 20 adult patients.
3. *Outcome measures*: the follow-up interval was 6 to 25 months. The primary endpoint is the incidence of target lesion revascularization (TLR). The secondary endpoint is the occurrence of a major adverse cardiovascular event (MACE). A MACE is mainly defined as target vessel revascularization (TVR), myocardial infarction (MI), all-cause death (ACM), and cardiac death (CD). Angiographic findings included minimum luminal diameter (MLD), late luminal loss (LLL), intrastent restenosis (BR), and percent diameter stenosis (DS%). When multiple follow-up events were reported, the outcome of the longest follow-up period was analyzed.
4. *Type of the study*: RCT or observational study.

### 2.2. Exclusion Criteria

The exclusion criteria for this study are as follows:

1. Non-Chinese and non-English literature
2. Duplicate published articles or earlier reports of the same outcome in the same study
3. Conference abstracts, letters, case reports, editorials, or expert opinions
4. Data, incomplete data, or documents that cannot be extracted

### 2.3. Search Strategy

The two authors (Wen-Juan Xiu and Hai-Tao Yang) conducted systematic literature searches using PUBMED, MEDLINE, EMBASE, Cochrane Database, ClinicalTrials.gov, and Wanfang to collect data on RCTs and observational studies, as well as the retrieval time of the DEB and second-generation DES in coronary ISR until June 2017. The keywords used were (“Drug-eluting balloon,” OR “DEB,” OR “Drug-coated balloon,” OR “DCB”) AND (“Drug eluting stent,” OR “DES,” OR “everolimus eluting stent,” OR “EES,” OR “Xience,” OR “Promus,” OR “Zotarolimus eluting stent,” OR “ZES,” OR “Resolute”) AND (“in stent restenosis,” OR “ISR”).

### 2.4. Document Quality Evaluation and Data Extraction

Quality assessment of the retrieved literature was evaluated by the two authors (Wen-Juan Xiu and Hai-Tao Yang) based on preestablished assessment criteria. The data of the published articles were then summarized. Randomized controlled trials were extracted in a standardized format, and the details of the observational studies were taken and transformed into a standardized scale. In the event of a dispute, the authors assisted one another in coming to an agreement through mutual discussion or referral by a third author (Xiang Xie).

The two authors (Wen-Juan Xiu and Hai-Tao Yang) extracted the tables based on predesigned data. The authors then independently extracted and cross-checked the data, and in cases of a dispute, they assisted one another in coming to an agreement through mutual discussion or third parties (Xiang Xie). Data extraction included (1) the basic information included in the study, including the research topics, year of publication, first author, specific model of the DEB and DES, dual antiplatelet therapy (DAPT), MACEs, and end event; (2) the baseline characteristics of the study population, including the age, gender, and risk factors; and (3) the results of the outcome measures and indicators.

### 2.5. Definition of Endpoints

The primary endpoint was target lesion revascularization (TLR) at long-term follow-up. The secondary endpoints included major cardiovascular adverse events (MACEs), target vessel revascularization (TVR), myocardial infarction (MI), all-cause mortality (ACM), and cardiac death. The results of the angiography were minimum luminal diameter (MLD), late luminal loss (LLL), percent diameter stenosis (DS%), and stent restenosis (IR). When there were multiple follow-up time points when the outcome of the case was reported, the longest follow-up of the outcome of the incident situation analysis was used.

### 2.6. Statistical Analysis

Meta-analysis was performed using RevMan 5.3 software. On comparing the outcomes of patients with coronary artery ISR treated with the DEB versus second-generation DES, the risk ratio (RR) and its 95% confidence interval (CI) were used to assess the incidence of TLR, TVR, MI, all-cause mortality, and MACEs. The mean (M), tandard deviation (SD), and 95% confidence interval (CI) were used to assess the incidence of MLD and DS% rate and LLL. Heterogeneity testing between studies was conducted using the Cochran *Q* and *I*^2^ tests. *I*^2^ values of 25, 50, and 75% correspond to low, medium, and high levels of heterogeneity, respectively. For the expected heterogeneous nature of the studies, we first used random effects models to analyze the data. To further reconcile the heterogeneity among studies, sensitivity analyses were performed by observing the change of the effect index after removing individual study results one by one. Publication bias was assessed using a funnel plot.

## 3. Results

### 3.1. Literature Search Results

**Figure 1:**
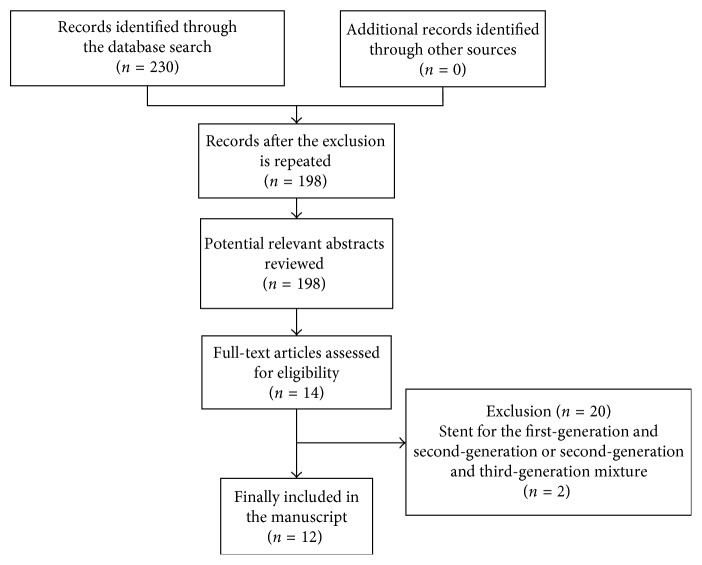
Flow diagram of the literature search and study selection.

A total of 230 articles were screened in the first screening. The articles were then screened out in layers, excluding review articles, duplicated literature, and those in which the authors failed to obtain the full text. A total of 12 articles were included in the final meta-analysis [14–24]. The literature search strategy and results are shown in Figure 1.

### 3.2. Characteristics of the Included Studies

**Figure 2:**
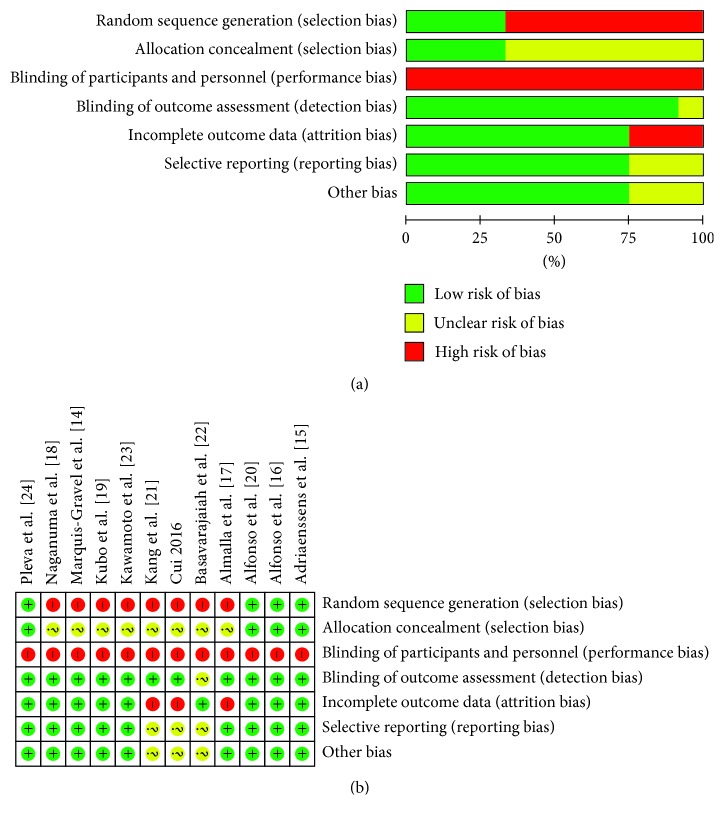
Document quality evaluation.

Four RCTs comparing the DEB versus second-generation DES [15, 16, 20, 24] including 684 patients and eight observational studies [14, 17–19, 21–23] including 1336 patients were included in the present study. One thousand patients with ISR were enrolled in the DEB group, and 1020 cases of ISR were included in the DES group. Three studies focused on BMS-ISR [15, 16, 24], five studies focused on DES-ISR [17, 20–22, 22], and two included BMS-ISR and DES-ISR [14, 23]. One study focused on the bifurcation of the ISR [18] via an ISR recursive treatment of the DEB [19]. Six studies [14, 17, 18, 21, 22] provided only clinical follow-up information and did not provide angiographic information. The clinical follow-up of each study ranged from 1 month to 5 years, and the follow-up results from 6 to 12 months were analyzed. The clinical characteristics of each study are shown in Tables 1–3. The quality of the included studies was acceptable. A flow chart of the quality assessment of the studies is shown in Figure 2.

### 3.3. Target Lesion Revascularization

**Figure 3:**
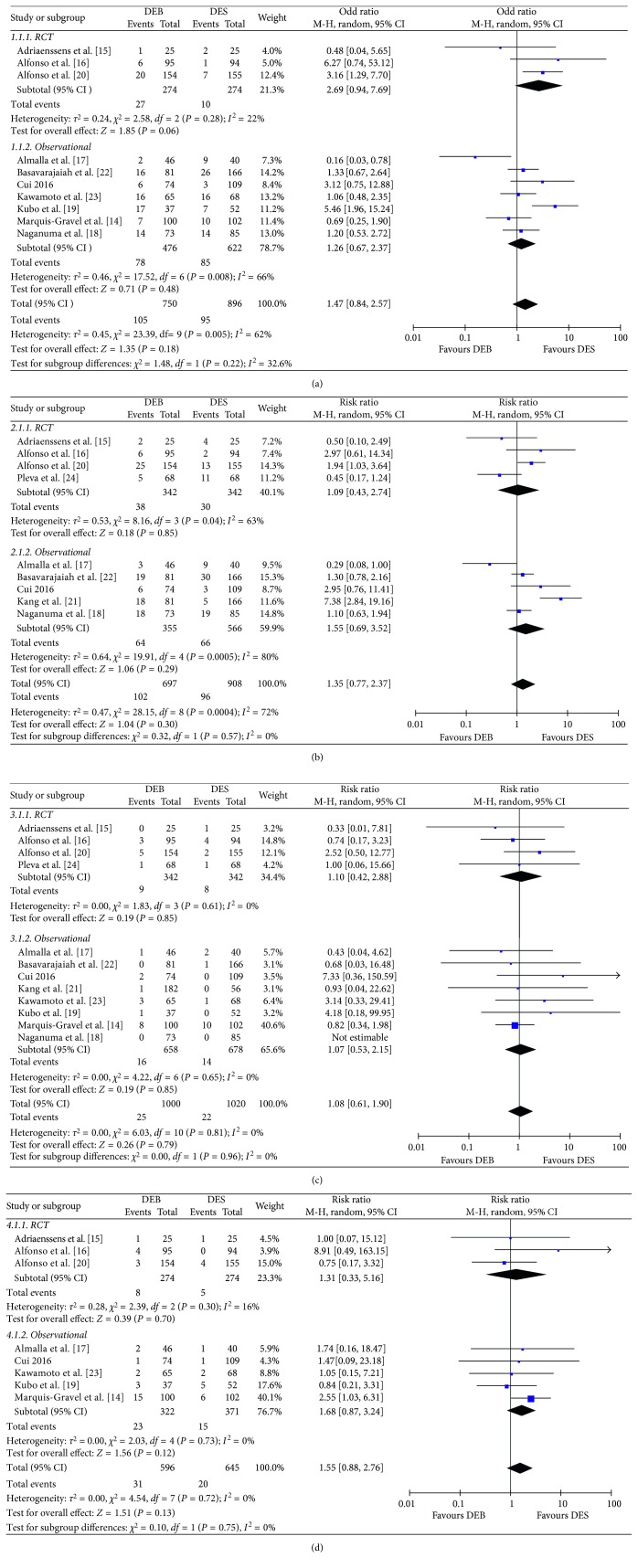
Clinical outcomes between the DEB group and DES group: (a) TLR; (b) TVR; (c) MI; (d) ACM.

As shown in Figure 3(a), ten [14–20, 22, 23] studies reported the incidence of target lesion revascularization. Meta-analysis suggested that there was no significant difference in the incidence of TLR (*P*=0.17) between the DEB group (14%) and DES group (10.6%). When only RCTs were considered, the heterogeneity of the results was lower (*I*^2^ = 22%; *P*=0.28). In the RCTs, the incidence of TLR in the DEB group had a tendency to increase, but the *P* value did not reach statistical significance (*P*=0.07). We did not find a significant difference in the incidence of TLR between the two groups in the observational studies (*P*=0.48).

### 3.4. Target Vessel Revascularization

As shown in Figure 3(b), nine studies [15–18, 20–22, 22, 24] reported the incidence of TVR at follow-up. There was no significant difference in the incidence of TVR (*P*=0.30) between the DEB group (14.6%) and DES group (10.5%) in either the RCTs or observational studies.

### 3.5. Myocardial Infarction

As shown in Figure 3(c), eleven [14–17, 19–24] studies reported the incidence of myocardial infarction at follow-up. We did not find a difference in the incidence of myocardial infarction between the DEB group (2.7%) and the DES group (2.3%; *P*=0.79).

### 3.6. All-Cause Mortality

As shown in Figure 3(d), eight [14–17, 19, 20, 22, 23] studies provided data on all-cause mortality at follow-up. There was no significant difference in the incidence of ACM between the DEB group (5.2%) and DES group (3.1%; *P*=0.13).

### 3.7. Cardiac Death

**Figure 4:**
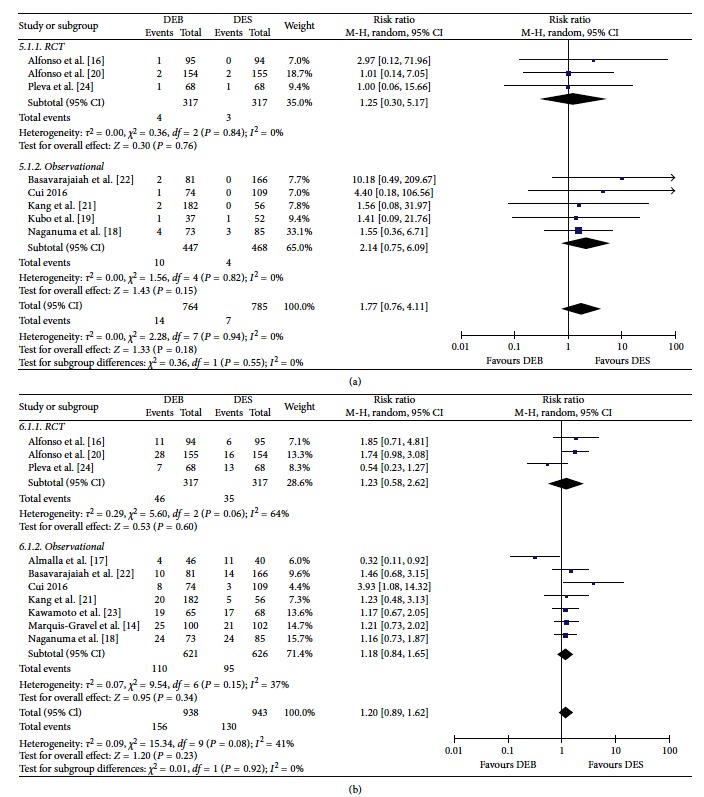
Cardiac death (a) and MACEs (b) between the DEB group and DES group.

As shown in Figure 4(a), eight [16, 18–22, 22, 24] studies provided the incidence of cardiac death at follow-up. The incidence of cardiac death in the DEB group demonstrated an increasing trend compared to the DES group; however, this result did not reach statistical significance (1.8% versus 0.9%; RR = 1.77; *P*=0.18).

### 3.8. Major Adverse Cardiovascular Events (MACEs)

As shown in Figure 4(b), 10 studies [14, 16–18, 20–24] provided the MACE incidence at follow-up. The overall incidence of MACEs between the DEB group (16.6%) and DES group (13.7%) was not significantly different (*P*=0.23). When only RCTs were considered, we also did not find significant difference in the incidence of MACEs when comparing the DEB group to the DES group (14.5% versus 11%; RR = 1.23; *P*=0.60).

### 3.9. Angiography Results

**Figure 5:**
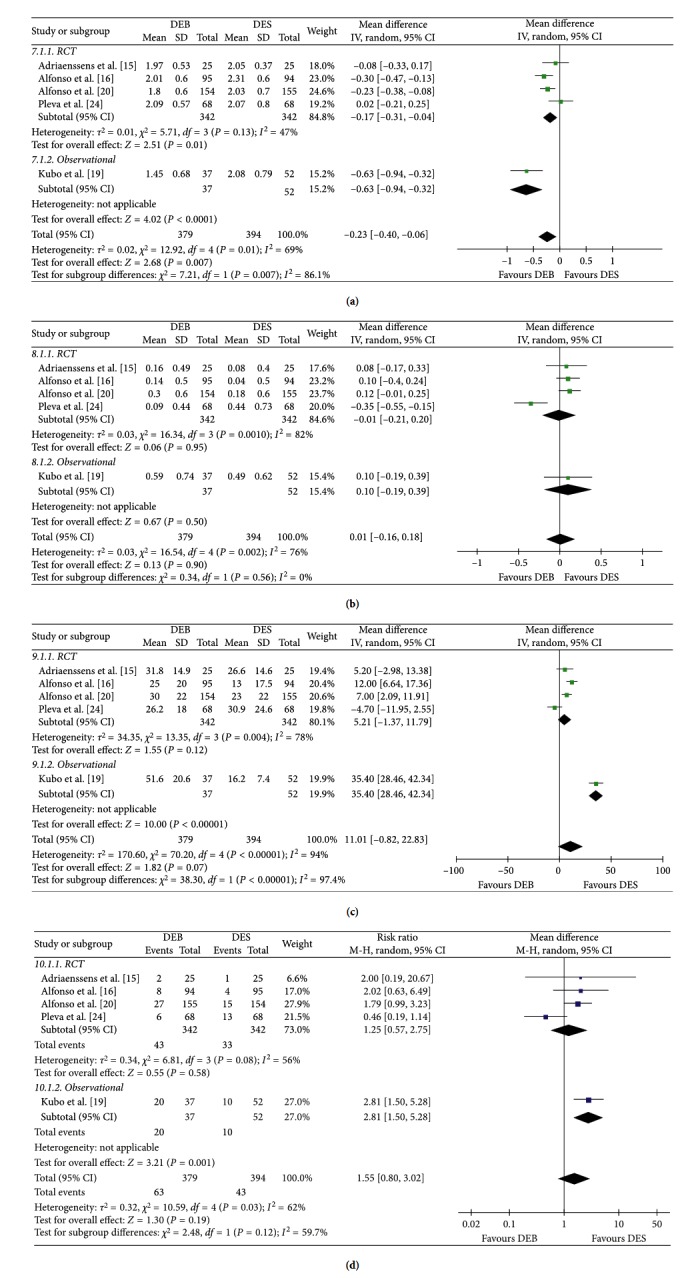
Coronary angiography outcomes between the DEB group and DES group: (a) MLD; (b) late loss; (c) binary restenosis; (d) DS%.

As shown in Figure 5, five studies [15, 16, 19, 20, 23, 24] provided angiography results. There was a statistically significant difference in the MLD between the DEB group and DES group (RR = 0.23; *P*=0.007). However, the incidence of late loss, binary restenosis, and DS% was not significantly different between the two groups.

### 3.10. Subgroup Analysis according to BMS-ISR and DES-ISR

The meta-analysis results suggested that, in DES-IRS but not in BMS-IRS, the difference in the MLD was significant. However, the incidence of TLR, TVR, MI, ACM, CD, MACEs, late loss, binary restenosis, and DS% was not significantly different between the DES group and DEB group (data not shown).

### 3.11. Sensitivity Analysis

We performed a sensitivity analysis to examine the influence of each study on the pooled RRs by removing each study one at a time. The pooled RRs showed no significant change, suggesting the results are stable.

To avoid some of the confounders present in the observational studies, we also excluded the observational studies and only analyzed the results of the RCTs. These results also showed no significant change, suggesting the results are stable.

### 3.12. Publication Bias Analysis

In the present study, we utilized funnel plots to evaluate the publication bias of all of the included studies. We did not find publication biases in this meta-analysis (data not shown).

## 4. Discussion

In this study, we performed a meta-analysis to compare the efficacy of the DEB to DES in the treatment of ISR. The present study suggests that, during 6–25 months of follow-up, the clinical outcomes are similar between the DEB group and DES group. This result suggests that the DEB is not inferior to the DES in the treatment of ISR.

In clinical practice, many treatment strategies have been developed for ISR patients after PCI, including POBA, cutting balloons, rotational atherectomy, and intravascular brachytherapy. However, most of these techniques have been replaced by the DES due to its side effect of inhibiting neointimal formation. Therefore, the DES has become the standard treatment for ISR. In addition, although there appears to be no evidence that the second-generation DES is superior to the first-generation DES [26], the second-generation DES is more biocompatible and its stent beam is thinner, thereby accelerating DES endothelialization and reducing neointimal formation [27]. However, CAD patients who were implanted with the DES required long-term dual antiplatelet therapy. In addition, reimplantation of the stent after ISR may result in reduced compliance of the coronary vessel wall and may damage branch opening. Furthermore, implantation of the stent may also cause an inflammatory response and stimulate the growth of endothelial tissue. The DEB allows for rapid and uniform release of the drug without the need for polymers and avoids reimplantation of the stent [28].

The literature published to date demonstrates that DEB treatment for BMS-ISR is very effective but is not as effective for the treatment of DES-ISR; in fact, the pathophysiology may be different. The metal in the stent stimulates the proliferation of blood vessels, and the polymer carrier on the surface of the drug stent also inhibits the repair of the vascular endothelium, resulting in the formation of a late thrombus. The drug-eluting balloon releases antiproliferative drugs locally to the vessel wall of coronary arteries, thereby achieving the effect of inhibiting intimal hyperplasia of the blood vessels and avoiding the need for additional stents and stent overlap, which also eliminates the increase of the intracoronary metal load. However, there are potential complications associated with the DEB. Compared with the DES, the DEB has no polymer matrix and no residual metal skeleton, which can reduce intimal inflammation and greatly reduce the risk of thrombosis, shortening the time for dual antiplatelet therapy (only 1 to 3 months after DCB). However, DCB treatment avoids the introduction of foreign bodies, which can result in follow-up treatment. The drug-eluting balloon is also less likely to compromise the ISR's involvement of the bifurcation's collaterals and may be more suitable for complex anatomies where stent implantation may not be ideal for drug delivery, such as curved or calcified blood vessels.

Persistent metal skeletons may remain the basis for stent thrombosis and restenosis. In recent years, endovascular neovascularization found in endoluminal imaging has confirmed this concept. In addition, the perpetuating metal skeleton has a risk of fracture, leading to adverse events, and the permanent influence of the metal skeleton on the normal vasomotion function of the stent at stent implantation is also an important factor that can lead to long-term adverse events.

Although the DEB can effectively inhibit the intimal hyperplasia of blood vessels, it cannot overcome the elastic retraction of blood vessels, which plays an important role in restenosis. Therefore, the DEB cannot completely replace the DES, and additional clinical data are still needed. The BRS supports diseased blood vessels early after implantation and is completely degraded after the negative remodeling of blood vessels is completed. After degradation, the BRS can restore the normal physiological and vasomotor function of the blood vessels, reduce inflammation of the blood vessel wall, and remove its influence on side branch vessels. Following repeated interventional treatment of the same lesion, the BRS can also be compatible with magnetic resonance imaging. In addition, at long-term follow-up, the BRS can result in late lumen enlargement.

At present, the materials used to make the BRS are primarily polymers (PLA) and metals (magnesium and iron). The BRS constructed from polymers has a relatively mature manufacturing process, while the BRS made from metals is difficult to use in clinical applications due to problems such as its degradation rate and inflammatory reaction. The only degradable PLA scaffold that has undergone large-scale clinical research and has been CE-approved is Abbott's Absorb BVS. Since the clinical study was conducted in 2007, the ABSORB series of studies and various small-scale real-world registration studies have demonstrated good clinical efficacy and safety in regard to both clinical and angiographic results during an early follow-up period of 1 to 2 years.

However, the three-year results of the ABSORB II [29] study and ABSORB III [30] study published by the American Society of Cardiology Annual Conference (ACC) in 2017 at the 2016 Annual Meeting of the Transcatheter Cardiovascular Therapeutics (TCT) did not meet the researchers' expectations. The three-year results of the ABSORB II study showed that the Abbott BVS was not a superior predictor of vasodilation and failed to show noninferiority expectations in terms of late lumen loss. Furthermore, the results of device-specific composite endpoints, target vessel myocardial infarctions, and advanced/late-stage stent thrombosis were clearly at a disadvantage compared with the Abbott BVS. The 2-year results of the ABSORB III study showed that the target vessel-target lesion failure of the Abbott BVS was significantly higher than that of the XIENCE stent, which was primarily reflected in small vessel lesions.

In our meta-analysis, we did not find a significant difference in clinical outcomes between the DEB group and DES group. The clinical endpoints observed in our analysis may only indicate short-term follow-up results. Clinical outcomes, such as MI, TLR, all-cause mortality, cardiac death, and TVR, may change significantly over time. Therefore, the present results require a large register or more elaborate RCTs with an appropriate long-term follow-up for validation.

## 5. Limitations of This Study

First, in the present study, only the Chinese literature and English literature were included. Due to differences in the ISR types and specific interventions (DES type and DAPT time) among the study populations, there was a certain level of heterogeneity between the included studies. Second, the shorter follow-up period included in the study and smaller sample size can only increase the reliability of the evaluation results to a certain extent. Finally, the inclusion of studies that failed to consistently report results (TLR, TVR, MI, ACM, CD, and angiographic findings) limited our scope of analysis.

## Figures and Tables

**Table 1:** Characteristics of the included studies.

Trial (year)	Treatment and no. of patients (*n*)		BMS- or DES-ISR	Type of the device		Study type	DAPT protocol	CAG F/U	Clinical F/U	MACE definition	Endpoint
				DEB	DES						
Marquis-Gravel et al. [14]	100	102	Canadian all comers	Paclitaxel	2nd generation	Observational	NR	NR	15 months	Death (all), nonfatal MI, TLR	Restenosis, MACE, stroke/TIA
Adriaenssens et al. [15]	25	25	Belgium BMS	Paclitaxel	Everolimus	RCT	3 months for DEB	9 months	12 months	Death (all), MI, TVR	% of struts uncovered, DS%, LLL, MLD, MACE
							12 months for DES				
Alfonso et al. [16]	95	94	Spain BMS	Paclitaxel	Everolimus	RCT	3 months for DEB	9 months	12 months	CD, MI, TVR	Death (all), TLR, MACE
							12 months for DES				
Almalla et al. [17]	46	40	Germany DES	Paclitaxel	Everolimus	Observational	NR	NR	DEB: 25 months	Death (all), MI, TVR	MACE, TLR, ST, MACE rate
									DES: 22 months		
Naganuma et al. [18]	73	85	Italy bifurcation ISR	Paclitaxel	Everolimus/zotarolimus	Observational	NR	NR	23 months	CD, MI, TVR	TLR, MACE
Kubo et al. [19]	37	52	Japan recurrent ISR after DEB	Paclitaxel	Everolimus	Observational	3 months for PCB	6–8 months	24 months	NR	ACM, CD, nonfatal MI, ST, TLR, MLD
							12 months for DES				
Alfonso et al. [20]	154	155	Spain DES-ISR	Paclitaxel	Everolimus	RCT	3 months for DEB	6–9 months	12 months	CD, MI, TVR	MLD, MACE
							12 months for DES				
Kang et al. [21]	182	56	DES	SeQuent Please	Everolimus	Observational	1 month for DCB	NR	24 months	CD, nonfatal MI, TVR	MACE
							12 months for DES				
Basavarajaiah et al. [22]	81	166	DES	Paclitaxel	2nd generation	Observational	1 month for DCB	NR	12 months	CD, MI, TVR	Death (all), TLR, ST, MACE
							12 months for DES				
Kawamoto et al. [23]	65	68	BMS- or DES-ISR	In.Pact Falcon	2nd generation	Observational	1 month for DEB	NR	12–24 months	ACM, MI, TLR	ST, MACE
				Pantera Lux			12 months for DES				
Pleva et al. [24]	68	68	BMS	Paclitaxel	Everolimus	RCT	3 months for DEB	12 months (±2 months)	6 months, 12 months	ACM, any MI, AR	LLL, BR, ST, MACE
							6–12 months for DES				
Cui et al. [25]	74	109	DES	SeQuent Please	2nd generation	Observational	3 months for DEB	NR	12 months	CD, nonfatal MI, TVR	MACE, no-event survival rate, ACM, TLR
							12 months for DES				

DEB: drug-eluting balloon; DES: drug-eluting stent; BMS: bare-metal stent; ISR: in-stent restenosis; RCT: randomized controlled trial; DAPT: dual antiplatelet therapy; CAG: coronary angiography; F/U: follow-up; N/A: not applicable; MACE: major adverse cardiac event; CD: cardiac death; ACM: all-cause mortality; MI: myocardial infarction; ST: stent thrombosis; TVR: target vessel revascularization; TLR: target lesion revascularization; MLD: minimum luminal diameter; LLL: late lumen loss; PCB: paclitaxel-coated balloon.

**Table 2:** 

Study	Demographics			Risk factors (*n*)						Indications (*n*)			
	Cohort	Age	Male (*n*)	HTN	DM	Smoke	Dyslipidaemia	Previous MI	Previous CABG	UAP	SAP	NSTEMI	Silent ischaemia
Marquis-Gravel et al. [14]	Overall	65	145	65	145	NR	91	NR	NR	NR	NR	145	NR
Adriaenssens et al. [15]	DEB	67.6 ± 7.7	18	16	6	5	24	12	NR	5	13	1	6
	DES	64.2 ± 11	25	15	1	3	24	10	NR	5	17	1	2
Alfonso et al. [16]	DEB	67 ± 11	82	68	30	56	69	57	4	38	43	NR	14
	DES	64 ± 12	82	68	19	70	62	56	7	42	41	NR	11
Almalla et al. [17]	DEB	69.6 ± 9.6	38	37	18	14	NR	17	10	NR	NR	NR	NR
	DES	67.7 ± 10.8	28	34	14	21	NR	21	4	NR	NR	NR	NR
Naganuma et al. [18]	DEB	67.2 ± 10.4	67	52	29	5	54	34	14	17	56 (including silent ischaemia and SAP)	NR	NR
	DES	65.2 ± 10.1	74	61	32	6	69	45	17	14	71 (including silent ischaemia and SAP)	NR	NR
Kubo et al. [19]	DEB	69.7 ± 9.7	32	30	18	28	24	19	6	NR	NR	NR	NR
	DES	71.3 ± 8.8	41	41	26	36	37	28	6	NR	NR	NR	NR
Alfonso et al. [20]	DEB	66 ± 10	127	110	75	89	110	73	16	80	74 (including silent ischaemia)	NR	NR
	DES	66 ± 10	130	121	66	87	121	77	17	79	79 (including silent ischaemia)	NR	NR
Kang et al. [21]	DEB	63.1 ± 9.8	125	132	80	85	165	NR	NR	60	NR	NR	NR
	DES	59.5 ± 11.0	36	39	16	26	46	NR	NR	24	NR	NR	NR
Basavarajaiah et al. [22]	DEB	66.8 ± 9.0	73	58	38	7	59	30	25	NR	NR	NR	NR
	DES	65.7 ± 9.6	143	119	55	12	127	85	56	NR	NR	NR	NR
Kawamoto et al. [23]	DEB	64.9 ± 9.1	57	51	28	6	51	36	17	NR	NR	NR	NR
	DES	67.2 ± 8.9	63	54	28	9	54	42	27	NR	NR	NR	NR
Pleva et al. [24]	DEB	65.6 ± 10.9	43	NR	17	NR	NR	43	3	NR	23 (including STEMI)	24	3
	DES	65.5 ± 10.6	46	NR	18	NR	NR	41	6	NR	18 (including STEMI)	25	10
Cui et al. [25]	DEB	61.9 ± 9.0	56	56	39	35	38	25	6	7	NR	NR	NR
	DES	61.5 ± 9.5	82	68	43	50	47	32	2	11	NR	NR	NR

HTN: hypertension; DM: diabetes mellitus; MI: myocardial infarction; CABG: coronary artery bypass graft; UAP: unstable angina pectoris; SAP: stable angina pectoris; NSTEMI: non-ST elevation myocardial infarction.

**Table 3:** Baseline angiographic characteristics.

Study	Pre-MLD		Pre-DS%		Lesion length (mm)		Post-MLD		Post-DS%	
	DEB	DES	DEB	DES	DEB	DES	DEB	DES	DEB	DES
Adriaenssens [15]	0.98 ± 0.60	0.57 ± 0.37	67.7 ± 18.4	79.4 ± 13.5	NR	NR	2.13 ± 0.45	2.12 ± 0.51	26.6 ± 13	25.9 ± 16.8
Alfonso et al. [16]	1.02 ± 0.40	0.93 ± 0.4	61 ± 14	65 ± 13	13.7 ± 7	13.8 ± 6	2.16 ± 0.5	2.38 ± 0.5	19 ± 11	11 ± 11
Almalla et al. [17]	0.57 ± 0.30	0.51 ± 0.41	NR	NR	9 ± 5.2	12.3 ± 11	2.42 ± 0.36	2.5 ± 0.5	NR	NR
Kubo et al. [19]	0.96 ± 0.45	0.80 ± 0.47	67 ± 14.9	72.2 ± 15.1	16.7 ± 12.9	15.7 ± 8.2	2.02 ± 0.4 4	2.56 ± 0.54	31.8 ± 10.3	16.2 ± 7.4
Alfonso et al. [20]	0.79 ± 0.40	0.75 ± 0.40	69 ± 17	72 ± 15	10.4 ± 5.6	10.7 ± 5.4	2.1 ± 0.4	2.22 ± 0.5	18 ± 10	13 ± 11
Kang et al. [21]	0.80 ± 0.40	0.80 ± 0.60	71.7 ± 5.2	74.6 ± 9.2	19.5 ± 8.9	21.3 ± 11.8	2.2 ± 0.4	2.7 ± 0.4	20.6 ± 11.9	13.6 ± 10.5
Kawamoto et al. [23]	0.74 ± 0.49	0.66 ± 0.43	74.8 ± 15.8	81.2 ± 14.4	18.7 ± 14.6	16.1 ± 9.6	2.34 ± 0.54	2.65 ± 0.48	18.2 ± 8.6	13.8 ± 7.6
Pleva et al. [24]	0.92 ± 0.45	0.79 ± 0.48	71.8 ± 13.9	78 ± 13.4	NR	NR	2.18 ± 0.39	2.51 ± 0.38	19.5 ± 7.4	16.3 ± 8.9

MLD: minimum luminal diameter; DS%: percent diameter stenosis; LLL: late lumen loss.

## References

[1] Dangas G. D., Claessen B. E., Caixeta A., Sanidas E. A., Mintz G. S., Mehran R. (2010). In-stent restenosis in the drug-eluting stent era. *Journal of the American College of Cardiology*.

[2] Rathore S., Terashima M., Katoh O. (2009). Predictors of angiographic restenosis after drug eluting stents in the coronary arteries: contemporary practice in real world patients. *EuroIntervention*.

[3] Stolker J. M., Kennedy K. F., Lindsey J. B. (2010). Predicting restenosis of drug-eluting stents placed in real-world clinical practice: derivation and validation of a risk model from the EVENT registry. *Circulation: Cardiovascular Interventions*.

[4] Farooq V., Gogas B. D., Serruys P. W. (2011). Restenosis: delineating the numerous causes of drug-eluting stent restenosis. *Circulation: Cardiovascular Interventions*.

[5] Intervention Cardiology Group of Chinese Medical Association Cardiology Branch (2016). Professional committees of cardiovascular clinic thrombosis prevention and control of Chinese Medical Association, Editorial Board of Chinese Journal of Cardiology. Guideline of Chinese percutaneous coronary intervention (2016). *Chinese Journal of Cardiology*.

[6] Han Y., Xu B., Jing Q. (2014). I-LOVE-IT 2 Investigators. A randomized comparison of novel biodegradable polymer- and durable polymer-coated cobalt-chromium sirolimus-eluting stents. *JACC: Cardiovascular Interventions*.

[7] Han Y., Xu B., Xu K. (2016). Six versus 12 months of dual antiplatelet therapy after implantation of biodegradable polymer sirolimus-eluting stent: randomized substudy of the I-LOVE-IT 2 trial. *Circulation: Cardiovascular Interventions*.

[8] Byrne R. A., Neumann F. J., Mehilli J. (2013). Paclitaxel-eluting balloons, paclitaxel-eluting stents, and balloon angioplasty in patients with restenosis after implantation of a drug-eluting stent (ISAR-DESIRE 3): a randomised, open-label trial. *The Lancet*.

[9] Xu B., Gao R., Wang J. (2014). A prospective, multicenter, randomized trial of paclitaxel-coated balloon versus paclitaxel-eluting stent for the treatment of drug-eluting stent in-stent restenosis: results from the PEPCAD China ISR trial. *JACC: Cardiovascular Interventions*.

[10] Lee J. M., Park J., Kang J. (2015). Comparison among drug-eluting balloon, drug-eluting stent, and plain balloon angioplasty for the treatment of in-stent restenosis: a network meta-analysis of 11 randomized, controlled trials. *JACC: Cardiovascular Interventions*.

[11] Femia G., Kushwaha V., Pitney M., Jepson N. (2013). Initial experience with drug-eluting balloons in the treatment of in-stent restenosis and de novo coronary lesions from two combined public and private cardiac catheterisation laboratories. *Heart, Lung and Circulation*.

[12] Mamuti W., Jiamali A., Rao F. (2014). Drug-coated balloon angioplasty for drug-eluting stent restenosis: insight from randomized controlled trials. *Ann Med*.

[13] Alazzoni A., Al-Saleh A., Jolly S. S. (2012). Everolimus-eluting versus paclitaxel-eluting stents in percutaneous coronary intervention: meta-analysis of randomized trials. *Thrombosis*.

[14] Marquis-Gravel G., Gobeil F., Noiseux N., Stevens L., Mansour S. (2013). Comparison of paclitaxel-eluting balloons with second-generation drug-eluting stents for treatment of in-stent restenosis: a retrospective analysis of an all-comers cohort. *Journal of the American College of Cardiology*.

[15] Adriaenssens T., Dens J., Ughi G. (2014). Optical coherence tomography study of healing characteristics of paclitaxel-eluting balloons vs. everolimus-eluting stents for in-stent restenosis: the SEDUCE (Safety and Efficacy of a Drug elUting balloon in Coronary artery rEstenosis) randomised clinical trial. *EuroIntervention*.

[16] Alfonso F., Pérez-Vizcayno M. J., Cárdenas A. (2014). A randomized comparison of drug-eluting balloon versus everolimus-eluting stent in patients with bare-metal stent-in-stent restenosis: the RIBS V clinical trial (Restenosis Intra-stent of Bare Metal Stents: paclitaxel-eluting balloon vs. everolimus-eluting stent). *Journal of the American College of Cardiology*.

[17] Almalla M., Schröder J., Pross V., Marx N., Hoffmann R. (2014). Paclitaxel-eluting balloon versus everolimus-eluting stent for treatment of drug-eluting stent restenosis. *Catheterization and Cardiovascular Intervention*.

[18] Naganuma T., Latib A., Costopoulos C. (2016). Drug-eluting balloon versus second-generation drug-eluting stent for the treatment of restenotic lesions involving coronary bifurcations. *EuroIntervention*.

[19] Kubo S., Kadota K., Otsuru S. (2015). Everolimus-eluting stent implantation versus repeat paclitaxel-coated balloon angioplasty for recurrent in-stent restenosis lesion caused by paclitaxel-coated balloon failure. *EuroIntervention*.

[20] Alfonso F., Perez-Vizcayno M. J., Cardenas A. (2015). A prospective randomized trial of drug-eluting balloons versus everolimus-eluting stents in patients with in-stent restenosis of drug-eluting stents: the RIBS IV randomized clinical trial. *Journal of the American College of Cardiology*.

[21] Kang I. S., Shehata I., Shin D. H. (2016). Comparison between drug-coated balloon angioplasty and second-generation drug-eluting stent placement for the treatment of in-stent restenosis after drug-eluting stent implantation. *Heart Vessels*.

[22] Basavarajaiah S., Naganuma T., Latib A. (2016). Treatment of drug-eluting stent restenosis: comparison between drug-eluting balloon versus second-generation drug-eluting stents from a retrospective observational study. *Catheterization and Cardiovascular Intervention*.

[23] Kawamoto H., Ruparelia N., Latib A. (2015). Drug-coated balloons versus second-generation drug-eluting stents for the management of recurrent multimetal-layered in-stent restenosis. *JACC: Cardiovascular Interventions*.

[24] Pleva L., Kukla P., Kusnierova P., Zapletalova J., Hlinomaz O. (2016). Comparison of the efficacy of paclitaxel-eluting balloon catheters and everolimus-eluting stents in the treatment of coronary in-stent restenosis: the treatment of in-stent restenosis study. *Circulation: Cardiovascular Interventions*.

[25] Cui K., Lu S., Song X. (2016). Drug-eluting balloon versus second-generation drug-eluting stent for the treatment of drug-eluting stent in-stent restenosis: an observational study. *Journal of Cardiovascular & Pulmonary Diseases*.

[26] Higgins JP. T., Green S. (2011). *Cochrane Handbook for Systematic Reviews of Interventions Version 5.1.0*.

[27] Alfonso F., Pérez-Vizcayno M. J., Dutary J. (2012). Implantation of a drug-eluting stent with a different drug (switch strategy) in patients with drug-eluting stent restenosis. Results from a prospective multicenter study (RIBS III [Restenosis Intra-Stent: Balloon Angioplasty versus Drug-Eluting Stent]). *JACC: Cardiovascular Interventions*.

[28] Cortese B., Micheli A., Picchi A. (2010). Paclitaxel-coated balloon versus drug-eluting stent during PCI of small coronary vessels, a prospective randomised clinical trial. The PICCOLETO study. *Heart*.

[29] Windecker S. (2016). *ABSORB STEMI-TROFI II–Two-Year Results, Transcatheter Cardiovascular Therapeutics (TCT) 2016*.

[30] Ellis S. (2017). *Everolimus-Eluting Bioresorbable Vascular Scaffolds in Patients with Coronary Artery Disease: ABSORB III Trial 2-Year Results*.

